# Supplementary material for: Distinct Cerebrospinal Fluid Lipid Signature in Patients with Subarachnoid Hemorrhage-Induced Hydrocephalus
Source: Biomedicines. 2023 Aug 23;11(9):2360. doi: 10.3390/biomedicines11092360 (PMC10525923; doi:10.3390/biomedicines11092360)
Supplement: Supplementary file 1 [file biomedicines-11-02360-s001.zip › biomedicines-2576643-supplementary.pdf]

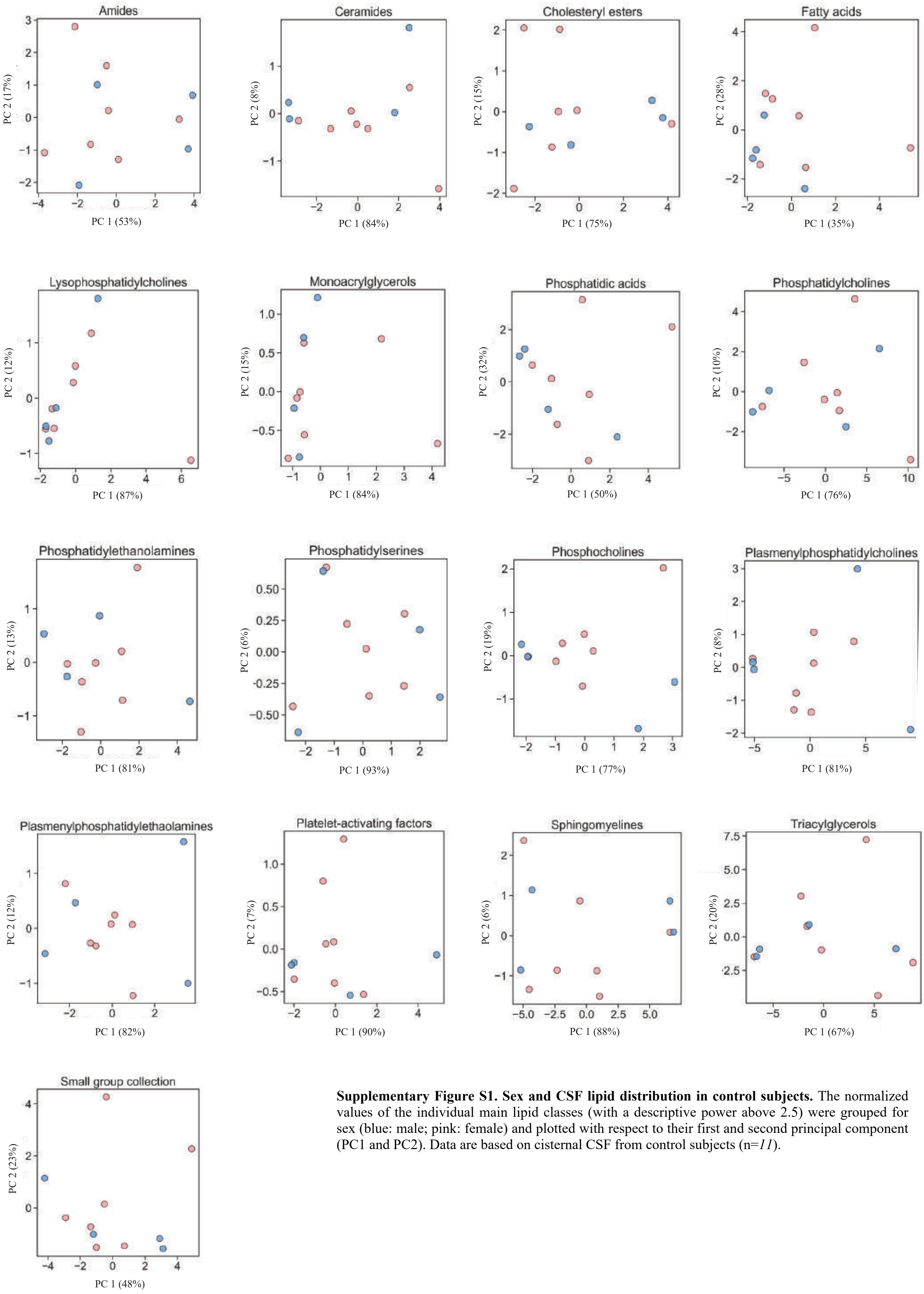

Supplementary Figure S1. Sex and CSF lipid distribution in control subjects. The normalized values of the individual main lipid classes (with a descriptive power above 2.5) were grouped for sex (blue: male; pink: female) and plotted with respect to their first and second principal component (PC1 and PC2). Data are based on cisternal CSF from control subjects (n=*11*).

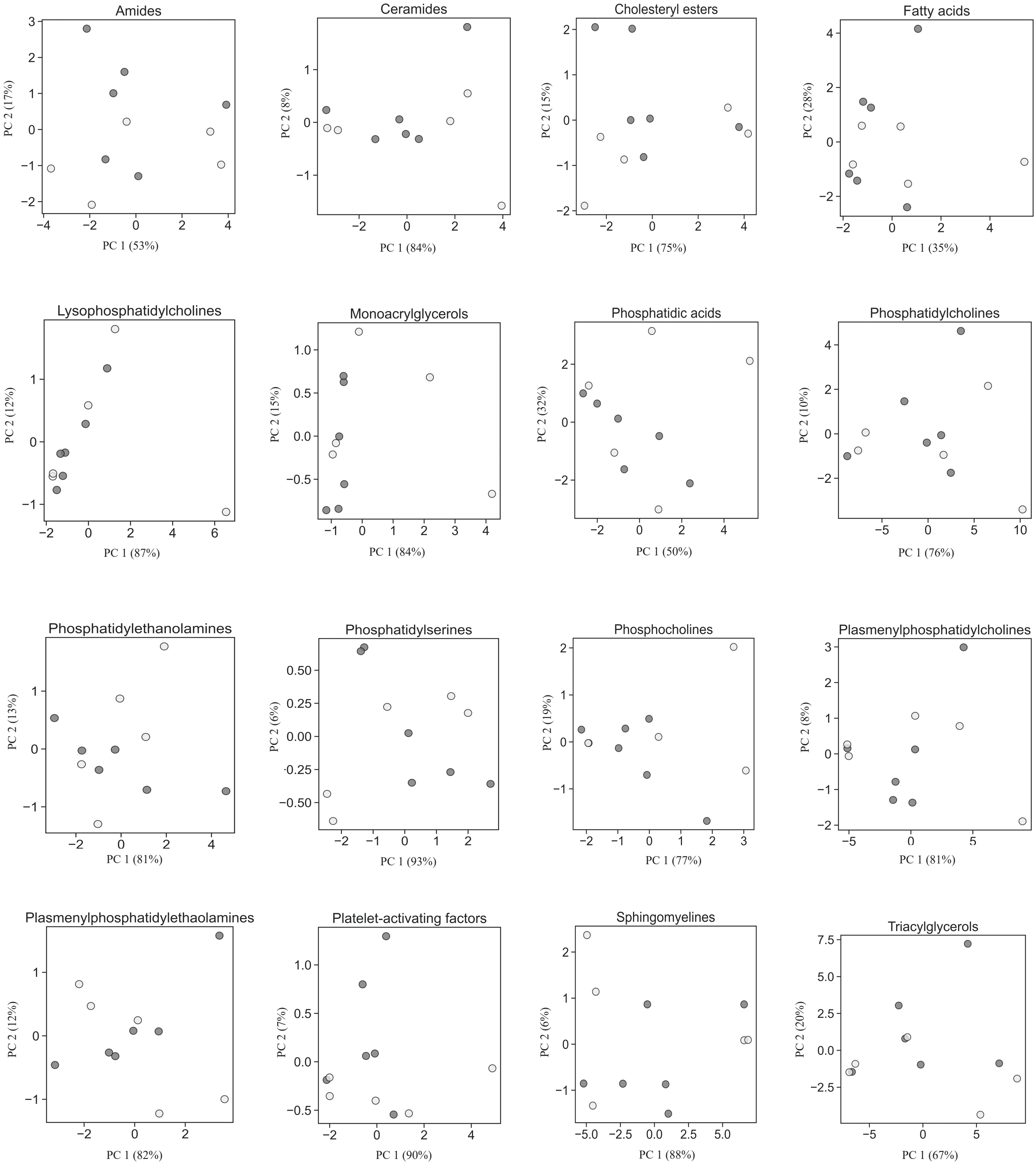

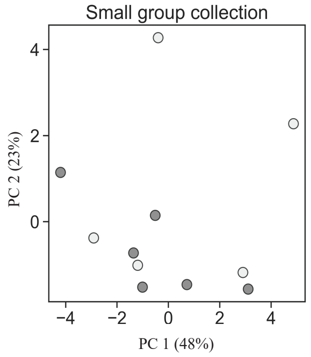

Supplementary Figure S2. Age and CSF lipid distribution in control subjects. The normalized values of the individual main lipid classes (with a descriptive power above 2.5) were grouped for age initially divided at the median age (light grey spheres: ≤ 60 years; dark grey spheres: > 60 years) and plotted with respect to their first and second principal component (PC1 and PC2). Data are based on cisternal CSF from control subjects (*n*=11).

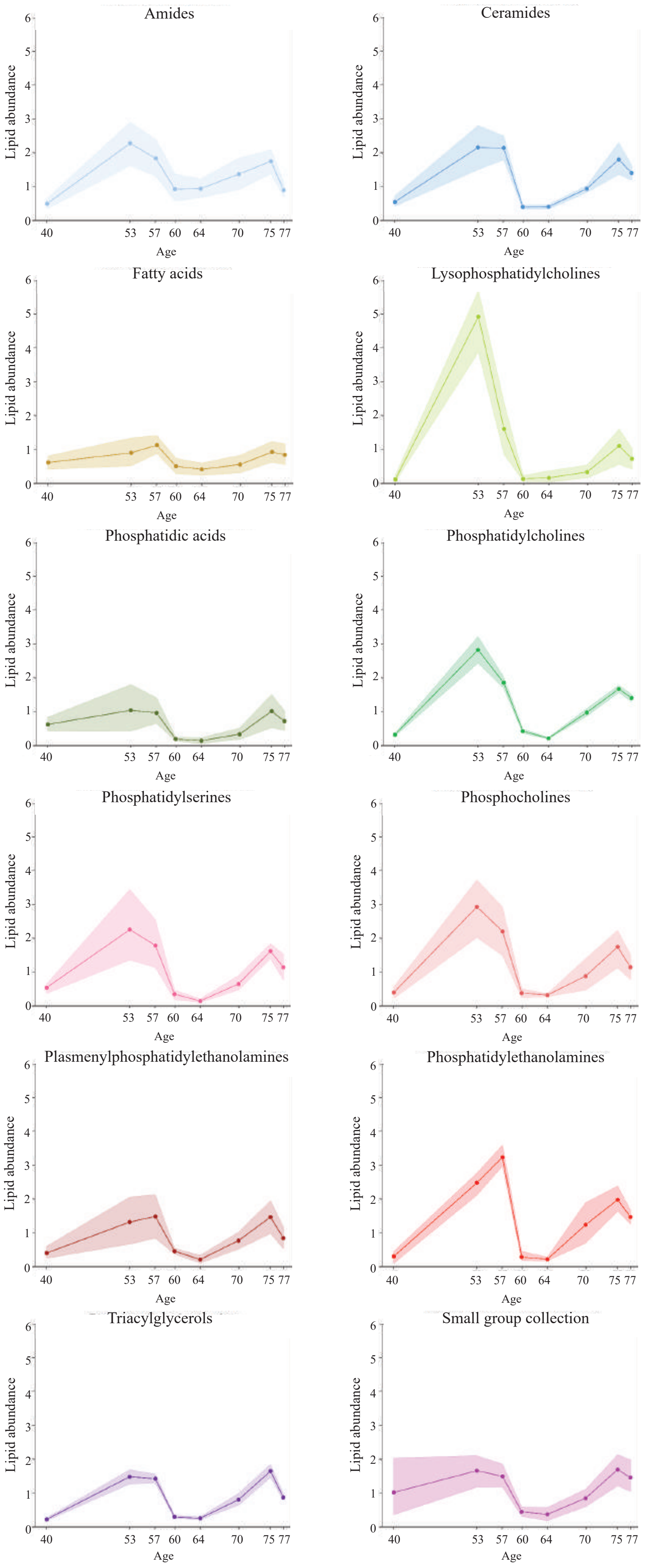

Supplementary Figure S 3. Lipid abundance fluctuation as a function of age. Individual lipid abundance fluctuation was generated based on normalized mean abundance for each lipid group as a function of the age of the control subjects and was plotted as a line plot from the youngest (40 years old) to the oldest (77 years old) control subject. Data are based on cisternal CSF from control subjects (*n*=11).

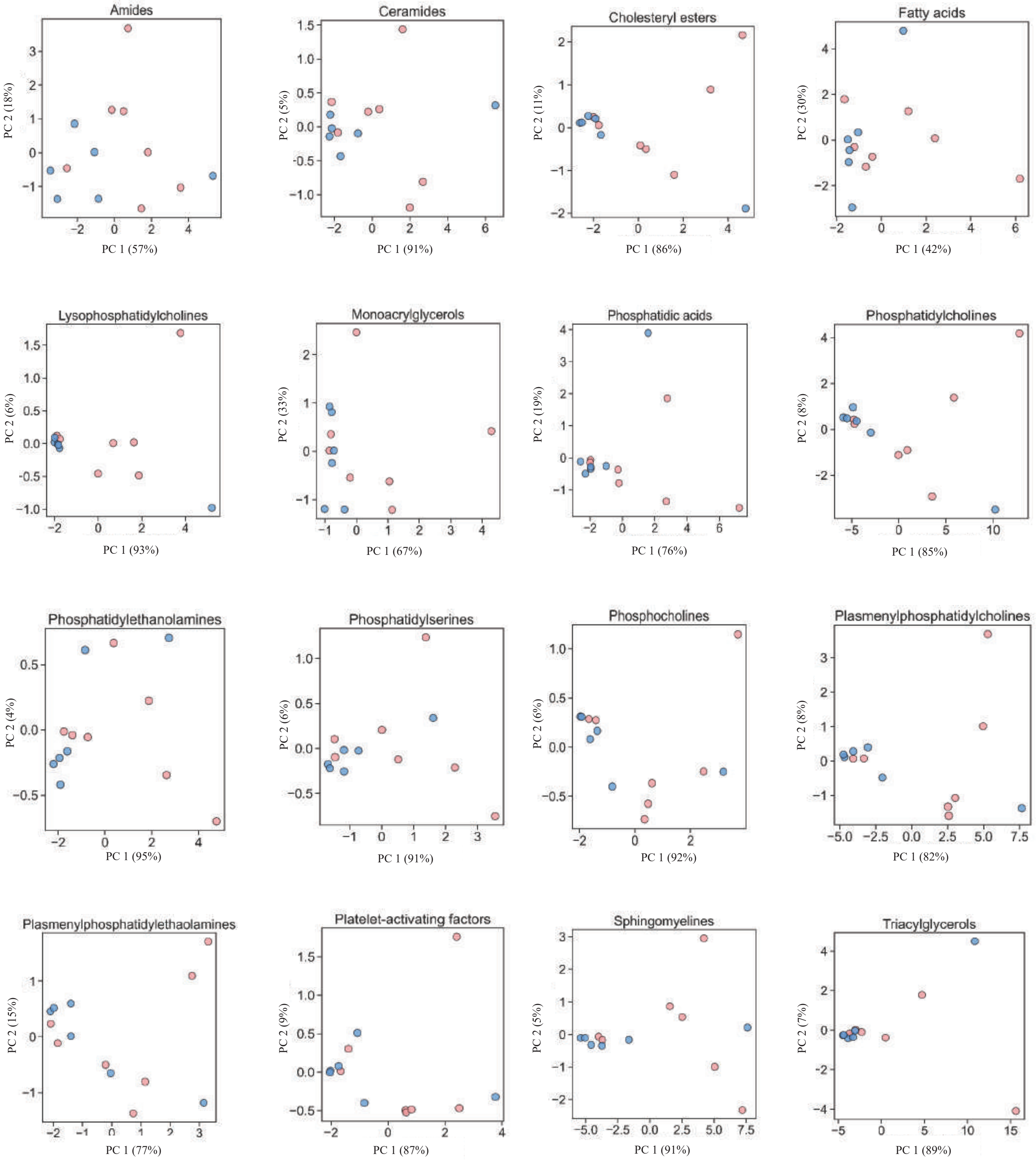

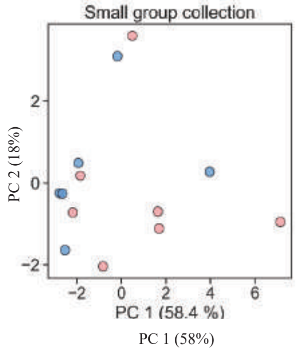

Supplementary Figure S4. Sex and CSF lipid distribution in patients with SAH. The normalized values of the individual main lipid classes (with a descriptive power above 2.5) were grouped for sex (blue: male; pink: female) and plotted with respect to their first and second principal component (PC1 and PC2). Data are based on cisternal CSF from patients with SAH (*n*=13).

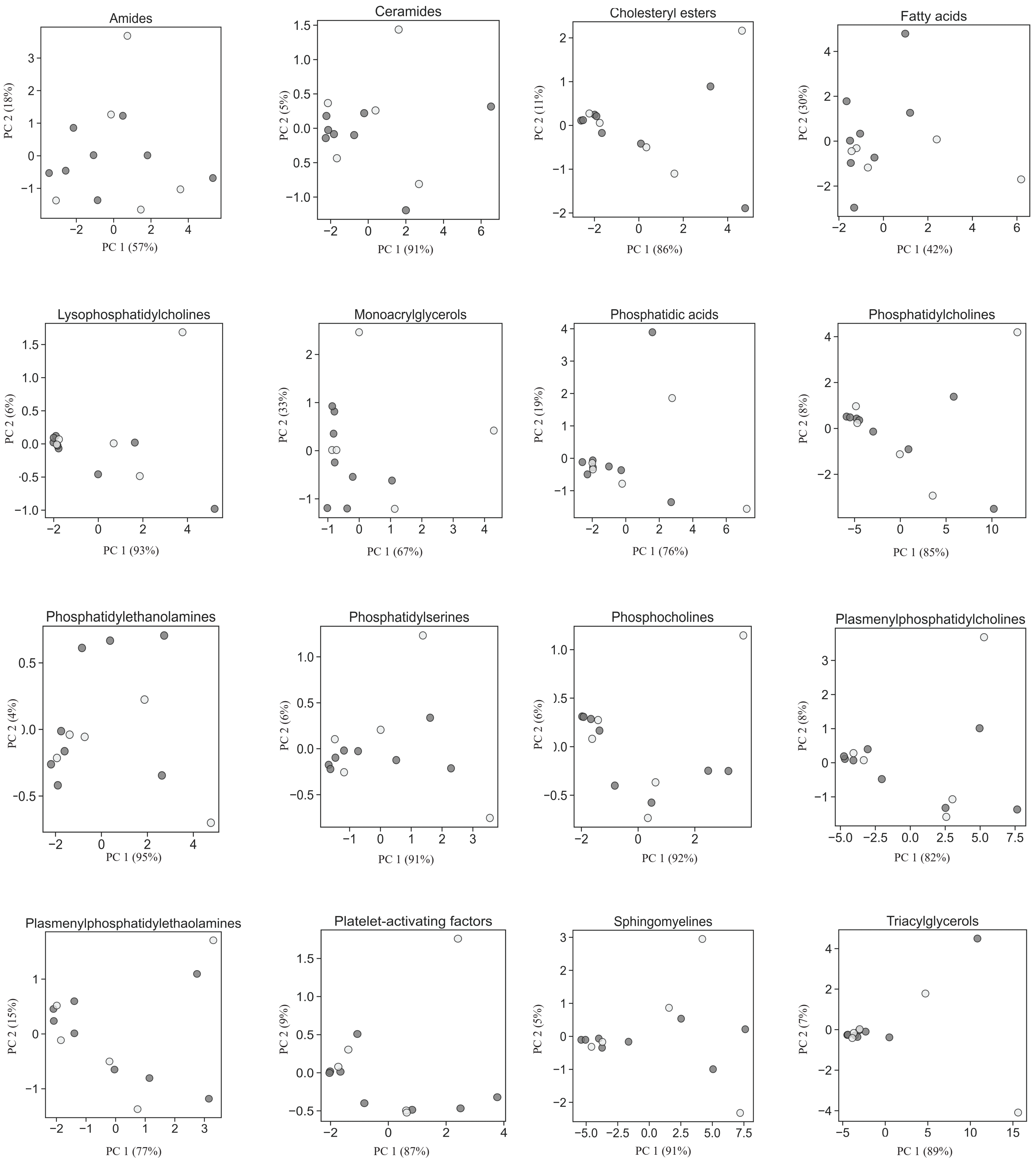

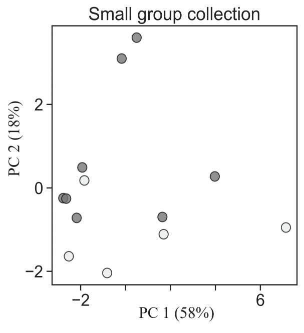

Supplementary Figure S 5. Age and CSF lipid distribution in patients with SAH. The normalized values of the individual main lipid classes (with a descriptive power above 2.5) were grouped for age initially divided at the median age (light grey spheres: ≤ 60 years; dark grey spheres: <60 years) and plotted with respect to their first and second principal component (PC1 and PC2). Data are based on cisternal CSF from patients with SAH (n=13).

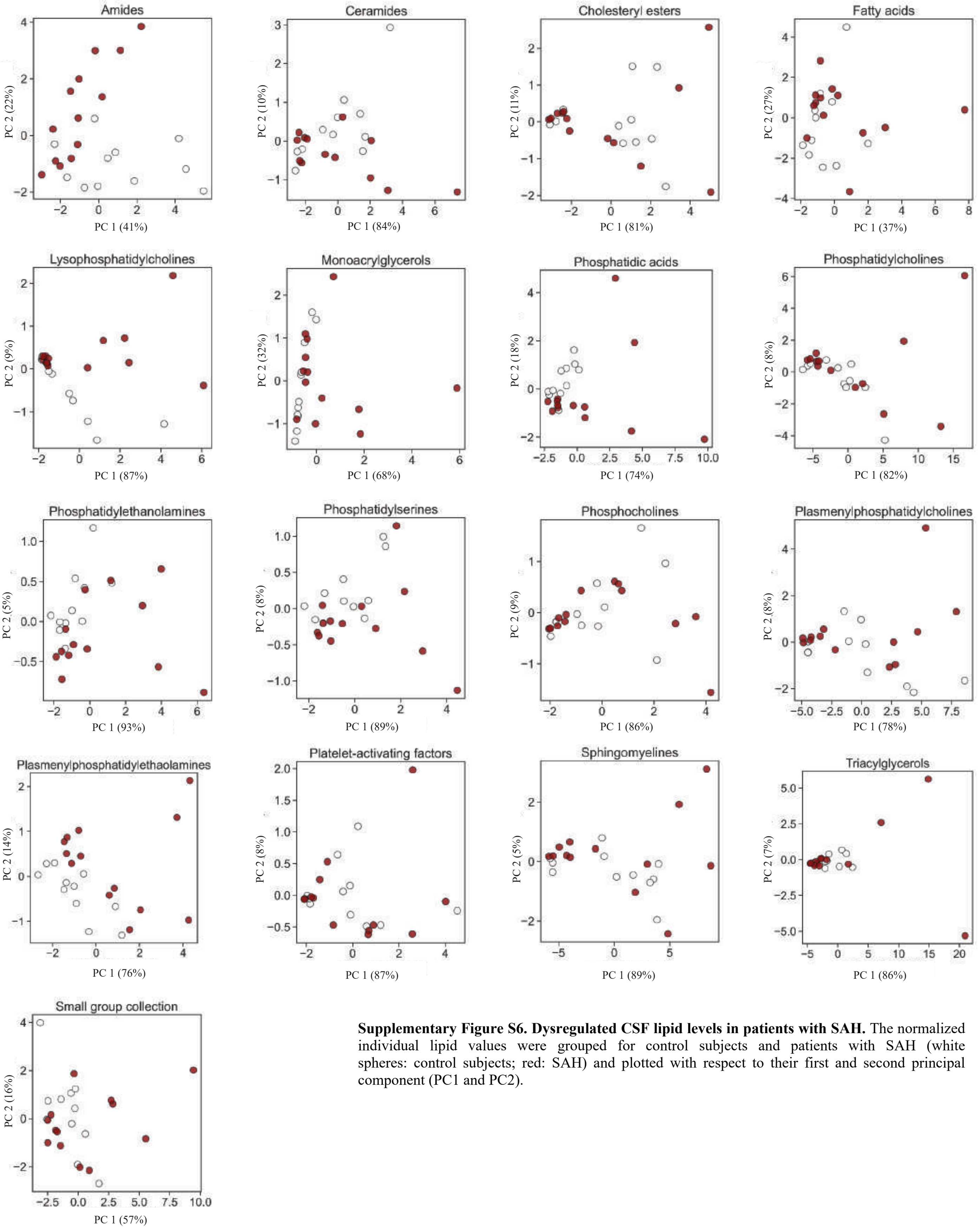

Supplementary Figure S6. Dysregulated CSF lipid levels in patients with SAH. The normalized individual lipid values were grouped for control subjects and patients with SAH (white spheres: control subjects; red: SAH) and plotted with respect to their first and second principal component (PC1 and PC2).

Table S1. Group overview: Overview of all lipids identified within the cerebrospinal fluid. Listed in the table is compound name together with t main class lipid group and the collected group (Groups) annotation used in this paper. Lipids of interest(LOI) is the lip are included in the data analysis (Yes) and the lipids which are excluded (no).

Compounds	Initial grouping	Groups	LOI
Oleamide	Amides	Amides	Yes
Z,14Z)-N-(2-Phenoxyethyl)-5,8,11,14-icosatetra	Amides	Amides	Yes
(9Z,12Z)-N-Hydroxy-9,12-octadecadienamide	Amides	Amides	Yes
S,3R,4E,6R)-1,3,6-trihydroxy-4-octadecen-2-yl]p	Amides	Amides	Yes
Amide C18	Amides	Amides	Yes
Heptadecanoyl Ethanolamide	Amides	Amides	Yes
Heptadecanoyl Ethanolamide.1	Amides	Amides	Yes
Linoleamide	Amides	Amides	Yes
Linoleyl ethanolamide	Amides	Amides	Yes
Oleoylethanolamide	Amides	Amides	Yes
a-linolenoyl-2-[(8Z,11Z,14Z)-icosatrienoyl]-sn-gl	Amides	Amides	Yes
Tricosanoyl Ethanolamide	Amides	Amides	Yes
Nervonic ceramide	Ceramides	Ceramides	Yes
C23-Ceramide	Ceramides	Ceramides	Yes
C24:1 b-D-Galactosyl ceramide	Ceramides	Ceramides	Yes
Ceramide (d18:1/22:0)	Ceramides	Ceramides	Yes
Ceramide (d18:1/24:0)	Ceramides	Ceramides	Yes
Glucosylceramide (d18:1/22:0)	Ceramides	Ceramides	Yes
Glucosylceramide (d18:1/24:0)	Ceramides	Ceramides	Yes
Ce 16:1	Cholesteryl esters	Cholesteryl esters	Yes
Ce 18:1	Cholesteryl esters	Cholesteryl esters	Yes
Ce 18:2	Cholesteryl esters	Cholesteryl esters	Yes
Ce 18:3	Cholesteryl esters	Cholesteryl esters	Yes
Ce 20:3	Cholesteryl esters	Cholesteryl esters	Yes
Ce 20:4	Cholesteryl esters	Cholesteryl esters	Yes
Ce 20:5	Cholesteryl esters	Cholesteryl esters	Yes
Ce 22:6	Cholesteryl esters	Cholesteryl esters	Yes
Ethyl oleate	Fatty acids	Fatty acids	Yes
FA 14:0	Fatty acids	Fatty acids	Yes
FA 16:1	Fatty acids	Fatty acids	Yes
10-(4-Sulfophenyl) decanoic acid	Fatty acids	Fatty acids	Yes
Palmitelaidic acid methyl ester	Fatty acids	Fatty acids	Yes
)-2-(Hydroxymethyl)-4-(12-methyloctadecyl)-3-	Fatty acids	Fatty acids	Yes
gmasta-5,22-dien-3-yl (7Z,10Z,13Z)-7,10,13-hex	Fatty acids	Fatty acids	Yes
a,16beta)-16-Acetoxy-3,5,14-trihydroxybufa-20,	Fatty acids	Fatty acids	Yes
(5E,9Z)-6-Bromo-5,9-icosadienoic acid	Fatty acids	Fatty acids	Yes
(5E,9Z)-6-Bromo-5,9-tricosadienoic acid	Fatty acids	Fatty acids	Yes
(5Z)-7,10-Dihydroxy-5-tetradecen-8-ynoic acid	Fatty acids	Fatty acids	Yes
(9Z)-17-(Dimethylarsoryl)-9-heptadecenoic acid	Fatty acids	Fatty acids	Yes
2-(4-methyl-2-oxo-3-penten-1-yl)-3,4-dihydro-2	Fatty acids	Fatty acids	Yes
noyloxy)-2-propanyl (5Z,8Z,11Z,14Z)-5,8,11,14-	Fatty acids	Fatty acids	Yes
loxy)-2-propanyl (5Z,8Z,11Z,14Z,17Z)-5,8,11,14,	Fatty acids	Fatty acids	Yes
oyloxy)-2-propanyl (6Z,9Z,12Z,15Z)-6,9,12,15-oc	Fatty acids	Fatty acids	Yes
13-(Dimethylarsoryl)tridecanoic acid	Fatty acids	Fatty acids	Yes
2,3-Bis(octanoyloxy)propyl decanoate	Fatty acids	Fatty acids	Yes
2,3-Dihydroxypropyl stearate	Fatty acids	Fatty acids	Yes
3-(Octanoyloxy)-1,2-propanediyl didecanoate	Fatty acids	Fatty acids	Yes
6-bromo-5E,9Z-pentacosadienoic acid	Fatty acids	Fatty acids	Yes
3-Oxodocosanoic acid	Fatty acids	Fatty acids	Yes
lysoPC 16:0, PC(16:0/0:0)	Lysophosphatidylcholines	Lysophosphatidylcholines	Yes
lysoPC 18:2, PC(18:2(9Z,11Z)/0:0)	Lysophosphatidylcholines	Lysophosphatidylcholines	Yes
LysoPC 17:0	Lysophosphatidylcholines	Lysophosphatidylcholines	Yes
LysoPC 18:0	Lysophosphatidylcholines	Lysophosphatidylcholines	Yes
LysoPC 20:4	Lysophosphatidylcholines	Lysophosphatidylcholines	Yes
LysoPC 22:6	Lysophosphatidylcholines	Lysophosphatidylcholines	Yes
MG 15:1	Monoacrylglycerols	Monoacrylglycerols	Yes
MG 18:2	Monoacrylglycerols	Monoacrylglycerols	Yes
MG 20:2	Monoacrylglycerols	Monoacrylglycerols	Yes
MG 22:5	Monoacrylglycerols	Monoacrylglycerols	Yes

oxy-2-hydroxypentylidene)amino]-5-hydroxyhex	Amino acids	Others	No
Arg-Glu	Amino acids	Others	No
D-Isoleucyl-L-prolyl-L-tyrosyl-D-isoleucine	Amino acids	Others	No
leu-gln	Amino acids	Others	No
2-hydroxy-3-[(2-methoxyicosyl)oxy]propoxy}pho	Amino acids	Others	No
hydroxy-3-[(2-methoxyoctadecyl)oxy]propoxy}p	Amino acids	Others	No
decanoyloxy)-2-[(15Z)-15-tetracosenoyloxy]pro	Amino acids	Others	No
adienoyloxy]-3-[(9Z)-9-heptadecenoyloxy]prop	Amino acids	Others	No
sadienoyloxy]-3-[(9Z)-9-nonadecenoyloxy]propo	Amino acids	Others	No
6,19-Docosahexaenoyloxy]-3-[(9Z)-9-heptadece	Amino acids	Others	No
cosadienoyloxy]-3-(heptadecanoyloxy)propoxy]	Amino acids	Others	No
cosadienoyloxy]-3-(heptadecanoyloxy)propoxy](	Amino acids	Others	No
)-9-octadecenoyloxy]-3-(stearoyloxy)propoxy]p	Amino acids	Others	No
2R)-2-hydroxy-3-(palmitoyloxy)propoxy]phosph	Amino acids	Others	No
R)-2-hydroxy-3-(tridecanoyloxy)propoxy]phosp	Amino acids	Others	No
nonadecanoyloxy)-3-(stearoyloxy)propoxy]phos	Amino acids	Others	No
pro-gln	Amino acids	Others	No
pro-gln.1	Amino acids	Others	No
Val-Ser	Amino acids	Others	No
Nafronyl	Meds_clinic	Others	No
Avapro	Meds_clinic	Others	No
Butabarbital	Meds_clinic	Others	No
Dexfenfluramine	Meds_clinic	Others	No
DL-Atenolol	Meds_clinic	Others	No
Iohexol	Meds_clinic	Others	No
Marimastat	Meds_clinic	Others	No
Valyltyrosine	Meds_clinic	Others	No
Nelfinavir	Meds_clinic	Others	No
O,N-DIDESMETHYLTRAMADOL	Meds_clinic	Others	No
Paroxetine	Meds_clinic	Others	No
Penciclovir	Meds_clinic	Others	No
Tafluprost	Meds_clinic	Others	No
Tigecycline	Meds_clinic	Others	No
Tricaprilin	Meds_clinic	Others	No
valganciclovir	Meds_clinic	Others	No
Zalcitabine	Meds_clinic	Others	No
Palmitoylcarnitine	Others	Others	No
4-Dihydroxy-7,8-dimethoxy-1,4-benzoxazin-3-o	Others	Others	No
(5E,9Z)-farnesyl acetone	Others	Others	No
1-(beta-D-ribofuranosyl)thymine	Others	Others	No
Bilirubin	Others	Others	No
Bis(2-ethylhexyl)adipate	Others	Others	No
(17Z)-17-Icosene-2,9,11,19-tetrayn-1-ol	Others	Others	No
xido-19,23,25-trioxa-24lambda~5~-phosphaoct	Others	Others	No
y-26-oxido-21,25,27-trioxa-26lambda~5~-phosp	Others	Others	No
xy-26-oxido-21,25,27-trioxa-26lambda~5~-phos	Others	Others	No
(2E)-3-Cyano-2-methyl-2-propen-1-yl stearate	Others	Others	No
ecyloxy)-3-(phosphonooxy)-2-propanyl (9Z)-9-he	Others	Others	No
,5S,6R)-2,3,4,5,6-pentahydroxycyclohexyl]oxy}p	Others	Others	No
-1-yloxy]-3-(phosphonooxy)-2-propanyl (9Z,12Z	Others	Others	No
8,11,14,17-Icosapentaenoyloxy]-3-(phosphonoo	Others	Others	No
9-octadecenoyloxy]propyl 2-(trimethylammonio	Others	Others	No
-Nonadecadienoyloxy]-3-(phosphonooxy)propy	Others	Others	No
,16,19-Docosahexaenoyloxy]-3-(docosyloxy)pro	Others	Others	No
16,19-Docosahexaenoyloxy]-3-(docosyloxy)prop	Others	Others	No
xy-3-methoxypropyl 2-(trimethylammonio)ethy	Others	Others	No
,14Z)-5,8,11,14-icosatetraenoyloxy]propyl 2-(tri	Others	Others	No
,4,5,6-pentahydroxycyclohexyl]oxy}phosphoryl)	Others	Others	No
loxy)-3-hydroxy-2-propanyl (10Z,13Z,16Z)-10,13	Others	Others	No
icosanoyloxy)-3-hydroxy-2-propanyl (13Z)-13-d	Others	Others	No
noyloxy)-3-hydroxy-2-propanyl (13Z,16Z)-13,16	Others	Others	No
ryl)undecanoyl]oxy}-3-hydroxy-2-propanyl 13-(	Others	Others	No
-Hydroxy-3-(icosanoyloxy)-2-propanyl henicosa	Others	Others	No
roxy-3-[(11Z)-11-icosenoyloxy]-2-propanyl heni	Others	Others	No
thylhexadecyl]oxy}propyl (2R)-2-hydroxy-1,3-pr	Others	Others	No
-O-(hydroxy{[(1S,2R,3R,4S,5S,6R)-2,3,4,5,6-pent	Others	Others	No

-hex-2-ulopyranonosyl}oxy)-1,3-dihydroxypropy	Others	Others	No
-6-[(1R,2R)-1,2,3-trihydroxypropyl]-beta-L-threo	Others	Others	No
-hex-2-ulopyranon7osyl}oxy)-1,3-dihydroxyprop	Others	Others	No
cetamido-3,5-dideoxy-6-[(1R,2R)-1,2,3-trihydro	Others	Others	No
oylamino]-3-hydroxy-4,14-octadecadien-1-yl 2-(	Others	Others	No
-tetracosenoylamino]-4,14-octadecadien-1-yl 2	Others	Others	No
ydroxy-24-(hydroxymethyl)-9,19-cyclolanostan-	Others	Others	No
1-Hexadecanoylpyrrolidine	Others	Others	No
2-(2-Acetoxy-2-oxoethyl)-2-hydroxysuccinic acid	Others	Others	No
R,2R)-2-(Aminomethyl)-1-hydroxycyclohexyl]ph	Others	Others	No
3-Deoxy-D-glycero-D-galacto-2-nonulosonic Acid	Others	Others	No
3,5-bis[(1R,6R)-6-isopropyl-3-methyl-2-cyclohex	Others	Others	No
5,3'-Digeranyl-3,4,2',4'-tetrahydroxychalcone	Others	Others	No
thoxyphenyl)ethyl]-2-methylphenoxy}-3-[2-(2-m	Others	Others	No
Adenosine cyclic 3',5'-monophosphate	Others	Others	No
Adenosine monophosphate	Others	Others	No
Coprine	Others	Others	No
cytidine 5'-monophosphate	Others	Others	No
Daucosterol	Others	Others	No
Dehydrofalcarinone	Others	Others	No
Enterostatin	Others	Others	No
goralatide	Others	Others	No
Hydroxycitronellal diethyl acetal	Others	Others	No
isodesmosine	Others	Others	No
Luteic acid	Others	Others	No
E)-1,4-heptadien-1-yl]-5-hydroxy-2-(4-hydroxyp	Others	Others	No
1-Isopropyl citrate	Others	Others	No
(R)-alpha-Tocotrienol	Others	Others	No
Myristyl sulfate	Others	Others	No
N~2~-Tetradecanoyl-L-glutamine	Others	Others	No
N-Hexanoyl-L-histidine	Others	Others	No
Pentosidine	Others	Others	No
cholesteryl beta-D-glucoside	Sugars	Others	No
N-Acetylneuraminic acid	Sugars	Others	No
[(stearoyloxy)methyl]-3,5,8-trioxa-4-phosphaoct	Sugars	Others	No
[(palmitoyloxy)methyl]-3,5,8-trioxa-4-phosphao	Sugars	Others	No
[(palmitoyloxy)methyl]-3,5,8-trioxa-4-phosphah	Sugars	Others	No
8-Hydroxy-8-methyl-3,9-decadienoyl]-beta-D-gl	Sugars	Others	No
-2-(4Z,7Z,10Z,13Z,16Z,19Z-docosahexaenoyl)-sn	Sugars	Others	No
N-acetyl-9-O-acetylneuraminic acid	Sugars	Others	No
N-Acetyllactosamine	Sugars	Others	No
Sucrose	Sugars	Others	No
l-1-[(2R)-7,7,7-trifluoro-6-hydroxy-2-heptanyl]o	Vitamins	Others	No
(5Z,7E)-9,10-Secocholesta-5,7,10-triene	Vitamins	Others	No
(5Z,7E)-9,10-Secocholesta-5,7,10-triene.1	Vitamins	Others	No
(5Z,7E)-9,10-Secocholesta-5,7,10-triene.2	Vitamins	Others	No
lmitoyl-alpha-D-glucopyranosyl)oxy]-2-icosanyl	Vitamins	Others	No
5,19,23,27,31-octamethyl-2,10,14,18,22,26,30-	Vitamins	Others	No
PA 22:0	Phosphatidic acids	Phosphatidic acids	Yes
PA 24:0	Phosphatidic acids	Phosphatidic acids	Yes
PA 26:0	Phosphatidic acids	Phosphatidic acids	Yes
PA 28:0	Phosphatidic acids	Phosphatidic acids	Yes
PA 39:3	Phosphatidic acids	Phosphatidic acids	Yes
PA 43:4	Phosphatidic acids	Phosphatidic acids	Yes
PA 45:4	Phosphatidic acids	Phosphatidic acids	Yes
PA 48:4	Phosphatidic acids	Phosphatidic acids	Yes
PA 49:4	Phosphatidic acids	Phosphatidic acids	Yes
PA 50:5	Phosphatidic acids	Phosphatidic acids	Yes
PC 32:0, PC(16:0/16:0)	Phosphatidylcholines	Phosphatidylcholines	Yes
PC 30:0	Phosphatidylcholines	Phosphatidylcholines	Yes
PC 31:0	Phosphatidylcholines	Phosphatidylcholines	Yes
PC 33:0	Phosphatidylcholines	Phosphatidylcholines	Yes
PC 32:1	Phosphatidylcholines	Phosphatidylcholines	Yes
PC 34:1	Phosphatidylcholines	Phosphatidylcholines	Yes
PC 35:1	Phosphatidylcholines	Phosphatidylcholines	Yes
PC 36:1	Phosphatidylcholines	Phosphatidylcholines	Yes

PC 36:1.1	Phosphatidylcholines	Phosphatidylcholines	Yes
PC 38:1	Phosphatidylcholines	Phosphatidylcholines	Yes
PC 33:2	Phosphatidylcholines	Phosphatidylcholines	Yes
PC 34:2	Phosphatidylcholines	Phosphatidylcholines	Yes
PC 35:2	Phosphatidylcholines	Phosphatidylcholines	Yes
PC 36:2	Phosphatidylcholines	Phosphatidylcholines	Yes
PC 36:2.1	Phosphatidylcholines	Phosphatidylcholines	Yes
PC 38:2	Phosphatidylcholines	Phosphatidylcholines	Yes
PC 34:3	Phosphatidylcholines	Phosphatidylcholines	Yes
PC 34:3.1	Phosphatidylcholines	Phosphatidylcholines	Yes
PC 35:3	Phosphatidylcholines	Phosphatidylcholines	Yes
PC 36:3	Phosphatidylcholines	Phosphatidylcholines	Yes
PC 36:3.1	Phosphatidylcholines	Phosphatidylcholines	Yes
PC 38:3	Phosphatidylcholines	Phosphatidylcholines	Yes
PC 34:4	Phosphatidylcholines	Phosphatidylcholines	Yes
PC 35:4	Phosphatidylcholines	Phosphatidylcholines	Yes
PC 36:4	Phosphatidylcholines	Phosphatidylcholines	Yes
PC 37:4	Phosphatidylcholines	Phosphatidylcholines	Yes
PC 38:4	Phosphatidylcholines	Phosphatidylcholines	Yes
PC 38:4.1	Phosphatidylcholines	Phosphatidylcholines	Yes
PC 40:4	Phosphatidylcholines	Phosphatidylcholines	Yes
PC 36:5	Phosphatidylcholines	Phosphatidylcholines	Yes
PC 37:5	Phosphatidylcholines	Phosphatidylcholines	Yes
PC 38:5	Phosphatidylcholines	Phosphatidylcholines	Yes
PC 40:5	Phosphatidylcholines	Phosphatidylcholines	Yes
PC 36:6	Phosphatidylcholines	Phosphatidylcholines	Yes
PC 37:6	Phosphatidylcholines	Phosphatidylcholines	Yes
PC 38:6	Phosphatidylcholines	Phosphatidylcholines	Yes
PC 39:6	Phosphatidylcholines	Phosphatidylcholines	Yes
PC 39:6.1	Phosphatidylcholines	Phosphatidylcholines	Yes
PC 40:6	Phosphatidylcholines	Phosphatidylcholines	Yes
PC 38:7	Phosphatidylcholines	Phosphatidylcholines	Yes
PC 38:7.1	Phosphatidylcholines	Phosphatidylcholines	Yes
PC 40:7	Phosphatidylcholines	Phosphatidylcholines	Yes
PC 40:8	Phosphatidylcholines	Phosphatidylcholines	Yes
PE 36:2	Phosphatidylethanolamines	Phosphatidylethanolamines	Yes
PE 36:4	Phosphatidylethanolamines	Phosphatidylethanolamines	Yes
PE 38:4	Phosphatidylethanolamines	Phosphatidylethanolamines	Yes
PE 38:6	Phosphatidylethanolamines	Phosphatidylethanolamines	Yes
PE 40:6	Phosphatidylethanolamines	Phosphatidylethanolamines	Yes
PS 39:0	Phosphatidylserines	Phosphatidylserines	Yes
PS 23:4	Phosphatidylserines	Phosphatidylserines	Yes
PS 39:7	Phosphatidylserines	Phosphatidylserines	Yes
PS 39:8	Phosphatidylserines	Phosphatidylserines	Yes
[(9Z)-hexadecenoyl]-sn-glycero-3-phosphocholi	Phosphocholines	Phosphocholines	Yes
adecyl-2-hexadecanoyl-sn-glycero-3-phosphoch	Phosphocholines	Phosphocholines	Yes
Z,10Z,13Z,16Z,19Z)-docosahexaenoyl]-sn-glycer	Phosphocholines	Phosphocholines	Yes
ctadecyl-2-arachidonoyl-sn-glycero-3-phosphoc	Phosphocholines	Phosphocholines	Yes
plasmenyl-PC 36:0	lasmenylphosphatidylcholin	Plasmenylphosphatidylcholines	Yes
plasmenyl-PC 34:1	lasmenylphosphatidylcholin	Plasmenylphosphatidylcholines	Yes
plasmenyl-PC 36:1	lasmenylphosphatidylcholin	Plasmenylphosphatidylcholines	Yes
plasmenyl-PC 42:1	lasmenylphosphatidylcholin	Plasmenylphosphatidylcholines	Yes
plasmenyl-PC 34:2	lasmenylphosphatidylcholin	Plasmenylphosphatidylcholines	Yes
plasmenyl-PC 36:2	lasmenylphosphatidylcholin	Plasmenylphosphatidylcholines	Yes
plasmenyl-PC 40:2	lasmenylphosphatidylcholin	Plasmenylphosphatidylcholines	Yes
plasmenyl-PC 42:2	lasmenylphosphatidylcholin	Plasmenylphosphatidylcholines	Yes
plasmenyl-PC 36:3	lasmenylphosphatidylcholin	Plasmenylphosphatidylcholines	Yes
plasmenyl-PC 38:3	lasmenylphosphatidylcholin	Plasmenylphosphatidylcholines	Yes
plasmenyl-PC 40:3	lasmenylphosphatidylcholin	Plasmenylphosphatidylcholines	Yes
plasmenyl-PC 36:4	lasmenylphosphatidylcholin	Plasmenylphosphatidylcholines	Yes
plasmenyl-PC 38:4	lasmenylphosphatidylcholin	Plasmenylphosphatidylcholines	Yes
plasmenyl-PC 40:4	lasmenylphosphatidylcholin	Plasmenylphosphatidylcholines	Yes
plasmenyl-PC 42:4	lasmenylphosphatidylcholin	Plasmenylphosphatidylcholines	Yes
plasmenyl-PC 44:4	lasmenylphosphatidylcholin	Plasmenylphosphatidylcholines	Yes
plasmenyl-PC 36:5	lasmenylphosphatidylcholin	Plasmenylphosphatidylcholines	Yes
plasmenyl-PC 38:5	lasmenylphosphatidylcholin	Plasmenylphosphatidylcholines	Yes

plasmenyl-PC 38:5.1	lasmenylphosphatidylcholin	Plasmenylphosphatidylcholines	Yes
plasmenyl-PC 40:5	lasmenylphosphatidylcholin	Plasmenylphosphatidylcholines	Yes
plasmenyl-PC 38:6	lasmenylphosphatidylcholin	Plasmenylphosphatidylcholines	Yes
plasmenyl-PC 40:6	lasmenylphosphatidylcholin	Plasmenylphosphatidylcholines	Yes
plasmenyl-PE 37:1	menylphosphatidylethaolam	Plasmenylphosphatidylethaolamines	Yes
plasmenyl-PE 36:4	menylphosphatidylethaolam	Plasmenylphosphatidylethaolamines	Yes
plasmenyl-PE 41:4	menylphosphatidylethaolam	Plasmenylphosphatidylethaolamines	Yes
plasmenyl-PE 40:6	menylphosphatidylethaolam	Plasmenylphosphatidylethaolamines	Yes
plasmenyl-PE 43:6	menylphosphatidylethaolam	Plasmenylphosphatidylethaolamines	Yes
Dihomo-I3-Linolenoyl PAF C-16	Platelet-activating factors	Platelet-activating factors	Yes
Dihomo-I3-Linolenoyl PAF C-16.1	Platelet-activating factors	Platelet-activating factors	Yes
Docosahexaenoyl PAF C-16	Platelet-activating factors	Platelet-activating factors	Yes
Eicosapentaenoyl PAF C-16	Platelet-activating factors	Platelet-activating factors	Yes
2,4-dihydroxyheptadec-16-ynyl acetate	Acetates	Small group collection	Yes
2,4-dihydroxyheptadec-16-ynyl acetate.1	Acetates	Small group collection	Yes
petroformynic acid B	Apolipoproteins	Small group collection	Yes
2H-Dibenzo[b,f]azepin-2-one	Bromides	Small group collection	Yes
3-(Methylthio)hexyl butanoate	Butanoic acids	Small group collection	Yes
-hydroxy-3-(3-methyl-2-butenyl)phenyl)-2-prop	Butyric acids	Small group collection	Yes
DG 36:3	Diacylglycerols	Small group collection	Yes
DG 36:4	Diacylglycerols	Small group collection	Yes
DG 38:4	Diacylglycerols	Small group collection	Yes
18-mercaptooctadecanoyl)-sn-glycerol 3-phosph	Lysophosphatidic acids	Small group collection	Yes
PG 33:2	Phosphatidylglycerols	Small group collection	Yes
enyl)-2-(9Z-octadecenoyl)-sn-glycero-3-phospho	Phosphoethanolamines	Small group collection	Yes
hexaenoyloxy]-2-[(1Z,11Z)-1,11-octadecadien-1-	Phosphoric acids	Small group collection	Yes
o-pentofuranosyl)-2,4-dioxo-1,2,3,4-tetrahydro-	Pyridines	Small group collection	Yes
Allopregnanolone	Steroids	Small group collection	Yes
Hydroxyprogesterone	Steroids	Small group collection	Yes
Nutriacholic acid	Steroids	Small group collection	Yes
oxido-11-oxo-12,16,18-trioxa-17lambda~5~-ph	Testosterone undecanoate	Small group collection	Yes
oxido-11-oxo-12,16,18-trioxa-17lambda~5~-ph	Testosterone undecanoate	Small group collection	Yes
-oxido-11-oxo-12,16,18-trioxa-17lambda~5~-ph	Testosterone undecanoate	Small group collection	Yes
Ricinolein	Triglycerides	Small group collection	Yes
SM 34:1, SM(d18:1/16:0)	Sphingomyelines	Sphingomyelines	Yes
SM 34:0	Sphingomyelines	Sphingomyelines	Yes
SM 36:0	Sphingomyelines	Sphingomyelines	Yes
SM 32:1	Sphingomyelines	Sphingomyelines	Yes
SM 33:1	Sphingomyelines	Sphingomyelines	Yes
SM 35:1	Sphingomyelines	Sphingomyelines	Yes
SM 36:1	Sphingomyelines	Sphingomyelines	Yes
SM 37:1	Sphingomyelines	Sphingomyelines	Yes
SM 38:1	Sphingomyelines	Sphingomyelines	Yes
SM 39:1	Sphingomyelines	Sphingomyelines	Yes
SM 40:1	Sphingomyelines	Sphingomyelines	Yes
SM 41:1	Sphingomyelines	Sphingomyelines	Yes
SM 42:1	Sphingomyelines	Sphingomyelines	Yes
SM 43:1	Sphingomyelines	Sphingomyelines	Yes
SM 32:2	Sphingomyelines	Sphingomyelines	Yes
SM 34:2	Sphingomyelines	Sphingomyelines	Yes
SM 36:2	Sphingomyelines	Sphingomyelines	Yes
SM 38:2	Sphingomyelines	Sphingomyelines	Yes
SM 40:2	Sphingomyelines	Sphingomyelines	Yes
SM 41:2	Sphingomyelines	Sphingomyelines	Yes
SM 42:2	Sphingomyelines	Sphingomyelines	Yes
SM 43:2	Sphingomyelines	Sphingomyelines	Yes
SM 44:2	Sphingomyelines	Sphingomyelines	Yes
SM 42:4	Sphingomyelines	Sphingomyelines	Yes
TG 48:2, TG(14:0/16:1/18:1)	Triacylglyceols	Triacylglycerols	Yes
TG 50:3, TG(16:1/16:1/18:1)	Triacylglyceols	Triacylglycerols	Yes
TG 50:4, TG(16:1/16:1/18:2)	Triacylglyceols	Triacylglycerols	Yes
TG 52:4, TG(16:0/18:2/18:2)	Triacylglyceols	Triacylglycerols	Yes
TG 56:6, TG(16:0/18:1/22:5)	Triacylglyceols	Triacylglycerols	Yes
TG 46:1	Triacylglyceols	Triacylglycerols	Yes
TG 46:2	Triacylglyceols	Triacylglycerols	Yes
TG 48:3	Triacylglyceols	Triacylglycerols	Yes

TG 51:3	Triacylglyceols	Triacylglycerols	Yes
TG 51:4	Triacylglyceols	Triacylglycerols	Yes
TG 50:5	Triacylglyceols	Triacylglycerols	Yes
TG 52:5	Triacylglyceols	Triacylglycerols	Yes
TG 53:5	Triacylglyceols	Triacylglycerols	Yes
TG 52:6	Triacylglyceols	Triacylglycerols	Yes
TG 54:6	Triacylglyceols	Triacylglycerols	Yes
TG 52:7	Triacylglyceols	Triacylglycerols	Yes
TG 54:7	Triacylglyceols	Triacylglycerols	Yes
TG 55:7	Triacylglyceols	Triacylglycerols	Yes
TG 58:7	Triacylglyceols	Triacylglycerols	Yes
TG 56:8	Triacylglyceols	Triacylglycerols	Yes
TG 58:8	Triacylglyceols	Triacylglycerols	Yes
TG 56:9	Triacylglyceols	Triacylglycerols	Yes
TG 58:9	Triacylglyceols	Triacylglycerols	Yes
TG 58:10	Triacylglyceols	Triacylglycerols	Yes
TG 58:11	Triacylglyceols	Triacylglycerols	Yes
TG 60:11	Triacylglyceols	Triacylglycerols	Yes
TG 51:2	Triacylglyceols	Triacylglycerols	Yes
TG 53:4	Triacylglyceols	Triacylglycerols	Yes
TG 51:5	Triacylglyceols	Triacylglycerols	Yes
TG 56:5	Triacylglyceols	Triacylglycerols	Yes
TG 55:6	Triacylglyceols	Triacylglycerols	Yes
TG 58:6	Triacylglyceols	Triacylglycerols	Yes
TG 56:7	Triacylglyceols	Triacylglycerols	Yes
TG 57:7	Triacylglyceols	Triacylglycerols	Yes
TG 54:8	Triacylglyceols	Triacylglycerols	Yes
TG 60:8	Triacylglyceols	Triacylglycerols	Yes
TG 60:9	Triacylglyceols	Triacylglycerols	Yes
TG 60:10	Triacylglyceols	Triacylglycerols	Yes
TG 62:10	Triacylglyceols	Triacylglycerols	Yes
TG 62:11	Triacylglyceols	Triacylglycerols	Yes
TG 62:12	Triacylglyceols	Triacylglycerols	Yes
TG 62:13	Triacylglyceols	Triacylglycerols	Yes
TG 46:3	Triacylglyceols	Triacylglycerols	Yes
